# Erleben von Zukunftsunsicherheit, Armutsrisiken und prekären Lebenslagen im Pflegeheim

**DOI:** 10.1007/s00391-023-02231-x

**Published:** 2023-09-20

**Authors:** Stefanie Richter

**Affiliations:** Fakultät für Angewandte Sozial- und Gesundheitswissenschaften, Ostbayerische Technische Hochschule (OTH Regensburg), Seybothstraße 2, 93053 Regensburg, Deutschland

**Keywords:** Altern, Hilfebedürftigkeit, Heimübergang, Armut, Autonomie, Ageing, Need of care, Home transition, Poverty, Autonomy

## Abstract

Die Situation von Menschen im Pflegeheim, die besonders dem Risiko der „pflegebedingten Verarmung“ ausgesetzt sind, ist in der Forschung wenig präsent. Der Beitrag befasst sich mit dem Erleben von Unsicherheiten, Armut und prekären Lebenslagen älterer Menschen im Heim. Grundlage liefern Daten einer ethnographischen Studie zum Älterwerden und zum Leben mit chronischen Erkrankungen. Anhand von Fällen wird ein Spektrum prekärer Lebenslagen skizziert. Deutlich wird u. a.: Menschen mit diversen soziobiografischen Hintergründen können im Zuge von Hilfebedürftigkeit und Heimübergängen von Unsicherheiten, Armut und prekären Lebenslagen betroffen sein. Nicht zu wissen, ob das verfügbare Geld genügt, um Kostensteigerungen bis zum Lebensende zu decken, kennzeichnet eine häufig erlebte Zukunftsunsicherheit; drohende Armut bzw. die Abhängigkeit von Sozialhilfe können pflege- und institutionell bedingte Verlusterfahrungen von Autonomie, Teilhabe und Lebensgestaltung verschärfen und den Gesamtzustand weiter destabilisieren. Das Erleiden bleibt weitgehend unsichtbar; strukturelle Probleme werden individualisiert.

## Abstract (en)

The living situation of people in nursing homes, with a specific vulnerability to care-related impoverishment, is underresearched. This article deals with experiences of uncertainty, poverty and a precarious life situation of care home residents in later life. The data basis is supplied by an ethnographic study of ageing and living with chronic health conditions. A range of precarious living situations are outlined on the basis of real cases. Insights gleaned include: people with diverse sociobiographical backgrounds can be affected by insecurity, poverty and precarious life situations as a result of becoming dependent on help and the transition to care homes. Not knowing whether available funds will suffice to cover rising costs until the end of life is a common form of uncertainty about the future. The threat of poverty or becoming dependent on state benefits can exacerbate experiences of loss associated with institutional care, losing autonomy, opportunities for participation, and options for shaping oneʼs life and can further destabilize the overall condition. This anguish remains largely invisible; structural problems are individualized.

Armut im Alter sowie die Folgen für die Lebens‑, Alltagsgestaltung und soziale Teilhabe werden vielfältig diskutiert. Studien zeigen, dass die Quote der Armutsgefährdung bei über 65-Jährigen steigt und über dem allgemeinen Durchschnitt liegt [1, 18]; dass immer mehr Menschen Grundsicherung im Alter erhalten, die Zahl der Anspruchsberechtigten sogar noch höher sei [1, 18]; dass Krankheit und Pflegebedürftigkeit das Armutsrisiko im Alter erhöhen [13] bzw. die Lebenslagen sozial benachteiligter Menschen verschärfen können [7]. Übersichtsbeiträge verweisen auf einen Bedarf an Studien, die sich mit dem Erleben, der Lebenswirklichkeit und den Umgangsweisen von älteren Menschen in Armut oder mit sozialer Ungleichheit und Pflege befassen [1, 10]. Wenige Arbeiten untersuchen biografische und strukturelle Konstellationen von Altersarmut [2, 3], beleuchten die Lebenssituation, Hilfearrangements und Teilhabe pflegebedürftiger Menschen bzw. die Bedeutung sozialräumlicher und individueller Ressourcen [8, 9, 5, 6]. Im Mittelpunkt stehen Menschen in ihrer Häuslichkeit. Das Thema Armut und prekäre Lebenslagen im Pflegeheim ist jedoch kaum präsent.

## Heimübergang und Armutsrisiken

Im Jahr 2021 erhielten 793.461 Menschen Leistungen der gesetzlichen Pflegeversicherung in Einrichtungen der stationären Langzeitpflege [4]. Die Zahl der Heime und Bewohner steigt kontinuierlich, wobei v. a. 80-Jährige und Ältere mit einem hohen Pflegegrad betroffen sind [16]. Das Risiko, ungeplant in ein Heim übersiedeln zu müssen, ist für viele ältere Menschen gegeben. Bereits 2005 fanden 37 % der Heimübergänge direkt vom Krankhaus (KH) statt [14], und Studien verweisen auf eine bedingte Einbeziehung der Betroffenen in die Entscheidung [11]. Menschen, die bereits vulnerabel sind, werden durch den (ungeplanten) Heimübergang mit komplexen Anforderungen und Verlusterfahrungen konfrontiert, die ihren Gesamtzustand gefährden können [11].

Mit dem Leben und der Pflege im Heim sind Kosten (Eigenanteile für Pflege, Unterkunft, Verpflegung, Investitions‑, Ausbildungskosten) verbunden, die für ältere Menschen und Angehörige zur Belastung werden können, auch weil der Anstieg des Eigenanteils schwer abzusehen ist. Im Jahr 2021 bezogen 36,80 % der Bewohner Hilfen zur Pflege nach dem Zwölften Sozialgesetzbuch (SGBXII, [13]). Ihr Vermögen genügt nicht, um die Kosten zu decken. Sie verfügen über wenig Spielraum, sich Dinge des täglichen Bedarfs zu leisten. Gemäß aktuellen Prognosen werden trotz politischer Bemühungen (Gesetz zur Weiterentwicklung der Gesundheitsversorgung, GVWG) der Eigenanteil und die Sozialhilfequote weiter steigen [13], und die „pflegebedingte Verarmung“ [13, S. 31] wird sich fortsetzen, was Deprivation, Scham, Abhängigkeit bzw. Ängste vor Armut fördert. Somit ist ein Heimübergang für viele Menschen nicht nur mit Einschnitten und Veränderungen in ihrem Alltag verbunden. Sie sind auch mit dem Risiko einer Abhängigkeit von Sozialhilfeleistungen konfrontiert, die zu (weiteren) Einschränkungen ihrer Entscheidungs‑, Gestaltungs‑, Teilhabechancen führen kann.

Das Ziel des vorliegenden Beitrags ist, prekäre Lebenslagen und Armutsrisiken im Heim aus der Erlebnisperspektive älterer Menschen darzustellen sowie Impulse für Forschung und Praxis zu geben. Die Autorin stieß im Rahmen ihrer ethnografischen Studie zum Älterwerden und zum Leben mit chronischen Erkrankungen wiederkehrend auf Themen wie finanzielle, materielle Not sowie das Erleben von Unsicherheiten und Kontrollverlusten, ohne dass sie dies forciert oder erfragt hatte.

## Grundlage: Studie zum Leben mit chronischen Erkrankungen im Alter

Ziele der Studie sind ein Verstehen der Lebenswelt älterer, chronisch erkrankter Menschen sowie die Rekonstruktion der soziobiografischen, sozialen und strukturellen Einbettung von Entwicklungs- und Bewältigungsprozessen. Angestrebt wird eine datengestützte Theoriegenerierung im Sinne der Grounded Theory [17] und der Narrationsanalyse [15].

Die Autorin führte 35 autobiografisch-narrative Interviews mit Menschen im Alter zwischen 57 und 94 Jahren, die mindestens ein dauerhaftes Gesundheitsproblem aufwiesen und sich in unterschiedlichen Wohn- und Lebenssituationen in Ost- und Westdeutschland befanden. Zugleich protokollierte sie Beobachtungen und Gespräche mit Angehörigen und Professionellen und erstellte dichte Beschreibungen der Interviewsituation und des Settings. Das Schreiben eines Memos diente der fortlaufenden Dokumentation, Strukturierung und Reflexion des Forschungsprozesses. Der Feldzugang und die Interviewauswahl erfolgten in Anlehnung an das Theoretical Sampling [17] sukzessiv: von der anfänglichen Offenheit mit wenigen Auswahlkriterien hin zu einer stärker theoretisch informierten Auswahl von Settings und Zielgruppen.

Zunächst erfolgte eine *Globalanalyse* [19] anhand der Protokolle, dichten Beschreibungen und Memos, um einen Überblick über die Daten zu gewinnen und um Phänomene offen zu kodieren und zu systematisieren. Im Ergebnis liegt eine über 300 Seiten umfassende Beschreibung von Phänomenen und deren Ausprägungen in den Einzelfällen vor, wie z. B. „Zukunftsunsicherheit“ und „prekäre Lage im Heim“. In der Globalanalyse wurden *der Übergang und das Leben im Heim* als einschneidende Erlebnisse mit komplexen und dauerhaften Bewältigungsanforderungen sowie die Wechselwirkungen zwischen biografischen, sozialen und strukturellen Konstellationen sichtbar [12]. Die Autorin entschied sich, die erste *Mikroanalyse* auf das Erleben und die Konstellationen des Übergangs und Lebens im Heim auszurichten und 16 der 35 Interviews, die in 5 Pflegeheimen und in einer Seniorenresidenz durchgeführt wurden, einzubeziehen.

In der Mikroanalyse wurde zunächst ein ‚Eckfall‘ je Einrichtung ausgewertet. Die Narrationsanalyse anhand des transkribierten Interviews dient der Rekonstruktion von Prozessstrukturen im Lebensablauf, von biografischen, sozialen und strukturellen Rahmungen sowie von Deutungs- und Verarbeitungsmustern unter Einbezug der dichten Beschreibungen, von Feldprotokollen sowie eines historisch informierten Wissens. Die intersubjektive Darstellung der Fallanalysen liegt in Form von Portraits vor (ca. 40 Seiten je Fall), aktuell je Einrichtung ein Portrait. Im 2. Schritt finden Fallvergleiche und Kontrastierungen statt, zunächst des Portraits mit den restlichen Fällen innerhalb der Einrichtung und anschießend zwischen den Einrichtungen zur Verdichtung und zur Erweiterung theoretischer Erkenntnisse. Die Autorin arbeitet zurzeit an den Fallvergleichen und bereitet eine Publikation vor.

In den Fallanalysen stieß sie erneut auf Phänomene wie materielle Not oder Unsicherheiten angesichts steigender Kosten im Zusammenhang mit Heimübergängen, wobei dies nicht nur Menschen betrifft, die objektiv als ‚einkommensarm‘ gelten. Es zeigen sich vielfältige Auswirkungen auf das Erleben und die Gesamtsituation der betroffenen Menschen.

Für den Beitrag hat die Autorin die Codes „Zukunftsunsicherheit“ und „prekäre Lage im Heim“ der Globalanalyse mit den dazugehörigen Fällen vertiefend analysiert. Einerseits hat sie die relevanten Portraits gesichtet, andererseits die dichten Beschreibungen, Feldprotokolle und Transkripte weiterer Fälle. Die Analyseschritte, Kurzdarstellungen zu den gesichteten Fällen mit Kommentaren und die Weiterentwicklung der theoretischen Erkenntnisse wurden dokumentiert.

## Spektrum erlebter Unsicherheiten, Armutsrisiken und prekärer Lebenslagen an Beispielen

Es werden mehrere Fälle skizziert, um das Spektrum an Beobachtungen abzubilden. Aus Platzgründen sind die Darstellungen sehr kondensiert und Zitate in begrenztem Umfang eingearbeitet. Auf weitere, sichtbar werdende Phänomene wie z. B. den Übergangsprozess kann nicht detailliert eingegangen werden. Personenbezogene Daten sind anonymisiert.

### Unsicherheiten und (selbstauferlegte) Beschränkungen in ‚gehobener‘ Wohnresidenz

Bei einer ‚gehobenen‘ Residenz wird davon ausgegangen, dass die älteren Menschen über genügend Vermögen verfügen. Das Beispiel zeigt, dass steigende Kosten angesichts begrenzter Ressourcen Verunsicherungen, (selbstauferlegte) Beschränkungen und Fremdheit befördern können, und dass das Setting zu einer Falle werden kann.

#### Frau Baldauf (85 Jahre alt), Abitur, verwitwet, ohne Familie, 70 % behindert, Pflegestufe 1, lebt seit 7 Jahren in einer Residenz.

Frau Baldauf war lange durch die Eltern, später durch ihren Mann abgesichert. Bis zur Ehe hatte sie Jobs als Tänzerin. Mit ihrem Mann genoss sie Urlaube und gutes Essen. Nach dem Tod des Gatten verfügt sie über eine Witwenrente und Ersparnisse. Sie möchte sich gut versorgt wissen und wählt eine Residenz. Sie nimmt das „kostengünstigste“ Apartment. Mit der Miete sind Mittagessen und 1 h Reinigung je Woche abgedeckt, jedes weitere Angebot kostet extra. Intern findet eine subtile soziale Differenzierung statt. An der Lage und Größe der Wohnung, an der Inanspruchnahme von Angeboten, an Alltagspraktiken oder am Auszug aufgrund der Kostenbelastung sind Vermögensverhältnisse und Milieu ablesbar. Fr. Baldauf fühlt sich von Beginn an fremd, hat kaum soziale Kontakte und hadert mit ihrer Situation. Das Finanzielle fällt ihr schwer, hatte sich doch ihr Mann darum gekümmert. Sie erlebt anhaltend Kostensteigerungen und meint: „Aber ich mache es mit Ach und Krach. Ich lass mir ja meine Pflegestufe auszahlen und alles zusammen. […] jede Hilfe muss ich extra bezahlen […] vermeide jedes Geld, was ich vermeiden kann.“ Sie beschreibt, wie sie Abendessen oder Veranstaltungen meidet; wie sie Arztbesuche scheut, denn sie muss den Begleitdienst zahlen (einschließlich Wartezeit); wie sie Medikamente „so lange strecke, wie nur möglich“. Zwar gibt es einen internen Pflegedienst, aber Bewohner ziehen offenbar aufgrund der steigenden Kosten immer wieder aus und in ein Heim. Sie erlebt wiederkehrend Niedergeschlagenheit; „Ich meine, Depressionen habe ich schon, häufig /hmh/ das ist immer dasselbe Thema, was viele hier haben, eben die *wahnsinns* Angst, bloß nicht ins Pflegeheim zu kommen, bloß nicht hilflos zu werden. […] denn natürlich denkt man immer, wenn man irgendwie, wenn’s, aber man weiß ja nie, wann es so weit ist und so. Man würde dann schon lieber was schlucken oder was, /hmh/, dass man gleich weg ist.“ Fr. Baldauf lebt zwar in einem gehobenen Setting, jedoch wird sie aufgrund ihrer wachsenden Hilfebedürftigkeit von absehbaren Kostensteigerungen bedroht. Sie bestreitet die Grundmiete über Rente und Pflegegeld und lebt ansonsten von begrenzten Ersparnissen. Sie vermeidet Kosten und zieht sich zurück. Ihre soziale Teilhabe ist beschränkt. Es bestehen Risiken der ‚Unsichtbarkeit‘ von Funktions- und Gesundheitseinbußen infolge von Kostenvermeidung sowie eines ungewünschten Heimübergangs aufgrund zu hoher Kosten. Ihre Ängste vor einem Heimübergang, ihre Hilflosigkeit und „Depressionen“ sind Ausdruck einer Gemengelage. Das gewählte Setting erweist sich bei ihr als Falle. Es repräsentiert ein Milieu, wo jeder über ausreichend finanzielle Mittel verfügt, seine Bedürfnisse artikuliert und bei Bedarf Serviceleistungen nachfragt. Die Absicherung durch die Familie im Lebensablauf zeigt außerdem ihre Tücke im Alter aufgrund niedriger Rentenansprüche und geringer Routine mit Verwaltungsangelegenheiten.

### Unsicherheiten, Verluste finanzieller Autonomie und prekäre Lebenslagen im Heim

Ein komplexer Versorgungsbedarf ist häufig nicht alleiniger *Grund für Heimübergänge.* Neben strukturellen Problemen (z. B. kürzere Liegezeiten im KH, Mangel ambulanter Alternativen) spielen *Finanzierungsgrenzen* (Wohnungsanpassung, Service usw.) eine Rolle. Zugleich bergen Übergänge *Risiken des Verlusts finanzieller Autonomie*, des Erlebens von Deprivation, Unsicherheit und weiteren Abhängigkeiten, wenn Rente und Erspartes den Eigenanteil nicht decken.

#### Frau Weinert (87 Jahre alt), Abitur, lebt seit 1,5 Jahren im Heim.

Frau Weinert hat in ihrem Leben einige Krisen bewältigt (z. B. plötzlicher Tod des selbstständigen Manns ohne Absicherung, Krebserkrankungen) und als alleinerziehende, berufstätige Mutter das Auskommen der Familie gesichert. Im Ruhestand wird sie künstlerisch aktiv, engagiert sich sozial, kulturell und pflegt soziale Kontakte. Unabhängigkeit, Selbstständigkeit und Gestaltungsspielraum sind für sie biografisch bedeutsame Werte. Sie kommt trotz begrenzter Geldmittel gut zurecht. Ihr Lebensentwurf sieht kein Lebensende im Heim vor. Als Funktionseinbußen den Alltag erschweren, engagiert sie den Sozialdienst auf eigene Kosten. Der Unterstützungsbedarf steigt, die Kosten werden unkalkulierbar, ihre Rente ist begrenzt, und sie möchte die Töchter nicht belasten. Das führt dazu, dass die Töchter der Mutter ein Heim empfehlen, das für sie finanzierbar ist. Trotz innerer Widerstände geht sie in das „kostengünstige“ Heim; sie weiß keine Alternative. Konfrontiert mit fremden Milieus und Ästhetiken zieht sie sich zurück. Zwar kann sie dank ihrer Töchter Praktiken wie Lesen (Lektüre) und Museumsbesuche aufrechterhalten, sie erlebt allerdings den Verlust von Sinn, Eigenständigkeit und sozialer Reziprozität: „Denn, äh, das is ja doch kein Leben mehr. Äh, ich muss im Leben muss ich was o:ch geben könn, was tun könn. /Hm/ Ja? Es, äh, es is=in Abwarten.“ Niedrige Rentenhöhe trotz Familien‑, Berufstätigkeit und Engagement sowie fehlendes Wissen um ambulante Alternativen bewirken einen nachhaltigen Bruch in ihrem Leben und ihren Freiheiten. Sie verbringt ihr Lebensende in einer Welt, die ihr fremd bleibt, und zieht sich zurück. Nach 1945 hatte sie mit ihrer Familie einen ‚sozialen Abstieg‘ erlebt, was sich in ihrem Erleben nun wiederholt.

#### Frau Buchmann (88 Jahre alt), mittlere Reife, Pflegestufe 2, lebt seit einem Jahr im Heim.

Frau Buchmann sorgte nach ihrer Scheidung für 3 Kinder und arbeitete bis zum 75. Lebensjahr, um ihren Lebensstandard zu sichern. Mit 80 zieht sie in ein altersgerechtes Wohnen in ihrem Kiez. Sie möchte unabhängig sein, sich die letzte Wohnstätte in gewohnter Umgebung schön gestalten und gibt dafür Ersparnisse aus. Nach einem Sturz wird sie im KH mit dem Heimübergang konfrontiert; Alternativen werden nicht geprüft. Die Kinder suchen ihr ein finanzierbares Zimmer, entfernt von ihrem Kiez, in der Peripherie mit wenig Anbindung an den öffentlichen Personennahverkehr (ÖPNV). Die Rente genügt nicht, sie muss auf ihre Ersparnisse zurückgreifen, neue Möbel zahlen. Die Vorstellung, dass das Geld nicht ausreicht und sie von den Kindern abhängig wird, belastet sie: „Meine Kinder versichern mir immer: ‚Mach Dir bloß keine Sorgen, wir ham alle Geld genuch‘ /Ja, klar/ besonders meine Jüngste, die unterstützt mich, die tut mir – (9 s) /hm/ (20 s) [weinend] + Also ich mach mir keine Sorgen + [weint] + /mm/ (8 s) /hm/ (7 s). Ach und allzu lange muss es ja auch nich’ mehr dauern +/mm/ (9 s)“. Sie wird nicht nur mit komplexen Verlusterfahrungen im Zuge des Sturzes und ungeplanten Heimübergangs konfrontiert, sondern auch mit finanziellen Abhängigkeiten. Im Interview hinterfragt sie wiederholend frühere Entscheidungen wie die eingereichte Scheidung, Urlaube mit Kindern bzw. das Einrichten des Apartments. Die Situation, nicht ausreichend Erspartes zu haben, führt zur kritischen Bewertung biografischer Entscheidungen als Fehler und gefährdet das Selbstbild einer unabhängigen Frau und Mutter.

Andere Bewohner, wie die folgenden Beispiele zeigen, sind plötzlich auf *Sozialhilfe und monatliche Budgets* von ca. 100 € angewiesen. Bei Professionellen und Bewohnern ist zu beobachten, dass „Taschengeld“ zur Alltagssprache gehört und Bewohner berichten, sich dieses ‚abholen‘ zu müssen („Kriegen wir auch nicht einfach so – müssen wir verlangen.“). Darin dokumentieren sich eine Normalisierung strukturell bedingter Abhängigkeiten, Asymmetrien und begrenzter Handlungsspielräume sowie eine Infantilisierung. Zudem scheinen Rechtsansprüche intransparent zu sein, und die Kontrolle über das eigene Geld scheint an ‚andere‘ überzugehen.

#### Frau Walther (89 Jahre alt), Abitur, Pflegestufe 1, verwitwet, seit 8 Mo. im Heim lebend.

Frau Walther wächst in Südamerika auf, migriert mit den Eltern im 2. Weltkrieg nach Deutschland, zieht 9 Kinder groß und arbeitet, da der Mann kriegsversehrt ist. Sie bewältigt den Tod des Gatten und eigener Kinder. Sie lebt bis 88 eigenständig, haushaltet mit begrenzten Mitteln und legt Wert auf ihr Äußeres. Sie spart Geld, um sich einen „roten Mantel“ zu kaufen. Nach einem körperlichen Zusammenbruch kommt sie direkt vom KH ins Heim. Sie ist in dieser Phase seelisch nicht dazu in der Lage, die Dinge zu ordnen. Kinder übernehmen die ‚Entsorgung‘ und eine Auswahl persönlicher Dinge, mit denen sie später im Heim aber wenig anfangen kann. Ihren „roten Mantel“ „schmeißen“ sie weg; dessen Verlust thematisiert sie wiederkehrend: „Ham die den guten Mantel weggeschmissen. Hab so lange dran gespart.“ Ihre Rente genügt nicht, die Kosten zu decken; sie muss Sozialhilfe beantragen: „Und da krieg ich bloß mein Taschengeld, ich bräuchte mal wieder ne Körperlotion, oder, n Haarwaschmittel und, muss ich alles selber kaufen. /hm/ Ich krieg nur Seife hier, aber damit kann ich doch nicht meine Haare, /hm/ und duschen damit und so, da brauch ich doch Duschdas und dis. (2 s) Das muss ich selber-, und denn das Katzenfutter /hm/ auch kaufen. Und das Streu für die Toilette, /hm/ (4 s). Da bleibt bloß n paar Euro übrig, hätte so gern mal n paar Apfelsinen, kann ich mir gar nicht leisten. Und hier is n Friseur /hm/. Für waschen und schneiden verlangt die 20 Euro. Das kann ich mir nicht leisten. […] Sehn Se mal [stöhnt] [leise] + Ich bin so matt + Wie finden Sie mich? Wie eine Mumie seh ich aus, nich’ /nein/ doch.“ Mit 89 Jahren erlebt sie, auf staatliche Hilfen angewiesen zu sein und sich nicht mal mehr das Nötigste für die Körperpflege und ihr Wohlbefinden leisten zu können. Zudem kann sie nicht auf für sie wertvolle Dinge zurückgreifen. Sie wurden entsorgt. Sie berichtet, sich aus Kostengründen zwischen dem Kauf von Katzenfutter oder einem Telefonat mit ihrer Schwester im Ausland entscheiden zu müssen.

#### Herr Ottmann (81), Grundschule, Pflegestufe 2, lebt seit 2 Jahren im Heim.

Herr Ottmann ist früh auf sich selbst gestellt, arbeitet ungelernt als Kind auf einem Bauernhof, anschließend im Bergbau und erlebt Unfälle, die ein Bein, den Kopf und die linke Hand dauerhaft beschädigen. Trotz Widrigkeiten gelingt es ihm, immer in Beschäftigung zu sein, sich eine eigene Existenz aufzubauen. Er ist fromm, legt viel Wert auf Ordnung und modische Kleidung. Im Ruhestand verdient er sich als „Toilettenmann“ Geld hinzu, um seinen Standard zu sichern. Ihm ist wichtig: „Und ick war *nie* ein Mensch, der vom Staat leben wollte.“ Er klärt frühzeitig seine Bestattung und legt Geld zurück. Er organisiert Putzhilfe, Mittagsdienst usw., um Einschränkungen zu kompensieren. Nach einem Schlaganfall mit 79 kommt er vom KH direkt ins Heim. Da seine Rente nicht genügt, ist er gezwungen, Sozialhilfe zu beantragen: „Aber jetzt nehm se alles weg. (2 s) /hm/ (3 s) Jetzt ham wa schon Sozialhilfe beantracht /hmh/, weil ick nur 30 Euro noch habe.“ Damit geraten auch seine Rücklagen für die Beerdigung in Gefahr „Und wenn sie die abheben, denn – /hm/ (3 s) /hm/, ick weiß nich (2 s). Ich seh es ja nachher nich, wo ick hinkomm. Aber, wat wolln Se machen. Könn Se nix dran ändern.“ Er erlebt neben komplexen Verlusten im Zuge des Schlaganfalls und plötzlichen Übergangs, die Kontrolle über seine Rente, Rücklagen sowie Lebensprinzipien zu verlieren. Mangels Systemwissen, kommunikativer Fähigkeiten, Fürsprecher und angesichts seiner Vulnerabilität steht er der Situation hilflos gegenüber. Er sitzt im Rollstuhl und kann ihn nicht allein nutzen (linke Hand fehlt, rechtsseitige Lähmung, Teppichboden), was nicht registriert wird. Seine Anfrage wegen eines elektronischen Rollstuhls bleibt ohne Antwort. Für die eigene Anschaffung fehlt das Geld. Gewohnte Ausflüge wie Spargelessen sind zu teuer. Er muss haushalten, auch um sich z. B. Seife oder einen Duschhocker kaufen zu können, stattdessen sind Kleidung und Möbel „uffn Mist jeschmissen“. Darin dokumentieren sich strukturell bedingte Beschränkungen, die Hr. Ottmann individuell verarbeiten muss.

#### Zuletzt Frau Gruber (75), Volksschule, Pflegestufe 2, seit 1,5 Jahren im Heim lebend.

Frau Gruber bewerkstelligt als („rumgestoßenet“) ‚Heimkind‘ ohne Ausbildung und Familie ihr Leben in der DDR alleinerziehend mit einem behinderten Kind. In den 1990er-Jahren verliert sie ihren Job. Trotz geringer Rente kommt sie in ihrer kleinen Wohnung klar. Am Monatsende isst sie „Stulle“, sodass etwas Geld bleibt und sie in die Stadt fahren und sich „Pullover oder Unterwäsche oder=n Rock“ kaufen kann. Ein Sturz und im KH entstandener Dekubitus führen dazu, dass sie gegen ihren Willen direkt ins Heim kommt. Sie erhält Sozialhilfe und „Taschengeld“. Sie kann sich wegen bestehender Schulden jedoch nichts leisten: „Neunzich Euro bleiben mir, bleiben mir /hmh/ und davon soll ick denn nu noch abzahln […] jetzt kann ick mir na nüscht mehr leisten nüscht, nüscht, na Essen und Trinken hab ick ja hier, naja Essen und Trinken, reicht ja o:ch, sach ick, aber ick wollt ma ja ma wat Neuet zum Anziehn wieder ma kaufen /hmh/ man möchte ja o:ch wieder wat Neuet ham, nich immer […] oder ich muss mit=n Becher in=e S‑Bahn jehn (2 s) mit=n Plastebecher [lacht leise], eine kleine Spende, oder ick muss unter de Bahnbrücke schlafen [lacht leise] (3 s) nee, a‑ da würd ick o:ch nüscht kriejen für /hmh/, vor allem weil ick ma=s nich traue, würd ma ja nich trauen [lacht leise] (3 s) ja.“ Die Schulden resultieren aus einem Servicevertrag mit einem Pflegedienst, den sie unwissend über Eigenanteile abgeschlossen hat. Sie weiß nicht, wie sie das Problem lösen und Bekleidungsgeld beantragen kann. Sie sucht Unterstützung beim Heimsozialdienst. Die Mitarbeiterin signalisiert ihr, überlastet zu sein und keine Zeit zu haben. Es gibt keine Fürsprecher; sie bleibt hilflos. Fr. Gruber fühlt sich fremd unter den älteren, hilfebedürftigen Menschen und erlebt einen Alltag ohne Anregung („na ja, is bisschen langweilig […] entweder gucken wa Fernsehen oder man jeht in Park, oder man kann hier o:ch uff de Straße“). Ihr Wunsch auf Rückkehr in die Häuslichkeit hat keine Chance, da ihre Wohnung aufgelöst und Ersparnisse für Möbel und Schulden aufgebraucht sind. Biografisch bedingt steht Fr. Gruber Institutionen eher ohnmächtig gegenüber. Es fehlt ihr an Wissen über ihre Rechte. Sie ist strukturell zum ‚Bleiben‘ gezwungen.

## Resümee

Deutlich wird, dass Menschen mit diversen soziobiografischen Hintergründen im Alter und im Zuge von Hilfebedürftigkeit und Heimübergängen von Unsicherheiten, Armut und prekären Lebenslagen betroffen sein können.

Sowohl bei Menschen im Heim als auch in der Häuslichkeit zeigt sich das Phänomen der *erlebten Zukunftsunsicherheit*, das sich aus verschiedenen Dimensionen speist:

- Wissen um begrenzte Rente bzw. Ersparnisse;
- Erleben steigender Lebenshaltungskosten;
- Wissen um das Risiko von Gesundheitseinbußen, Pflegebedürftigkeit und damit verbundenen Kosten;
- Ungewissheit über die verbleibende Lebenszeit und (weitere) eintretende Gesundheitsprobleme;
- biografische und soziale Hintergründe, die Ängste befördern.

Die Verunsicherung, ob das verfügbare Geld den antizipierten Kostenanstieg bis zum (unklaren) Lebensende genügt, kann sich auf den Gesamtzustand auswirken und verschiedene Umgangsweisen wie selbstauferlegte Beschränkungen, Vermeidung von Kosten bis hin zur kritischen Lebensrückschau hervorrufen.

Die Fälle zeigen außerdem,

a) dass Heimübergänge wiederkehrend ungeplant stattfinden und neben sozialem, kulturellem Kapital und Angebotsstrukturen materielle Ressourcen über den Verbleib in der bzw. die Rückkehr in die Häuslichkeit sowie über die Heimauswahl und Qualität des Übergangs entscheiden. Aus Kostengründen übersiedeln bzw. eine kostengünstige Variante wählen zu müssen, befördert die mit dem Heimübergang verbundenen Risiken, sich mit einer fremden Lebenswelt konfrontiert zu erleben;

b) dass die bereits vulnerablen Menschen neben pflege- und institutionell bedingten Abhängigkeiten weitere Verluste ihrer Autonomie, Lebensgestaltung und Teilhabe im Zuge von Sozialhilfe bzw. drohender Armut erleben, was ihren Gesamtzustand zusätzlich destabilisieren und das Selbstbild beschädigen kann. Die Zuweisung von „Taschengeld“ ist symbolisch für Asymmetrien, Abhängigkeiten und Infantilisierung;

c) dass die dargelegten Phänomene im Alltag weitgehend *unsichtbar* bleiben und *still erlitten* werden, einerseits wegen der Scham, der komplexen Belastungen der Betroffenen bzw. Individualisierung struktureller Probleme, andererseits mangels niedrigschwelliger Angebote, Diagnose bzw. verfügbarer/abrechenbarer Maßnahmen seitens Professioneller. Pflege und Soziale Arbeit böten wichtige Fürsprecher auf individueller, sozialer und politischer Ebene, wozu sie oftmals mangels fehlender Ressourcen nicht in der Lage sind.

d) dass ein Verstehen von Armut im Alter durch interpretative Zugänge wichtig wäre: Wie Menschen ihre schwierige Situation erleben, bewerten und damit umgehen, und welche Konstellationen Verunsicherung, Armutsrisiken und prekäre Lebenslagen befördern, lässt sich im soziobiografischen und strukturellen Zusammenhang nachvollziehen.

## Fazit für die Praxis

- Menschen mit diversen soziobiografischen Hintergründen können im Zuge von Hilfebedürftigkeit und Heimübergängen von Unsicherheiten, Armut und prekären Lebenslagen betroffen sein.
- Erlebte Zukunftsunsicherheit, drohende Armut bzw. die Abhängigkeit von Sozialhilfe können pflege- und institutionell bedingte Verlusterfahrungen von Autonomie, Teilhabe und Lebensgestaltung verschärfen und den Gesamtzustand der Betroffenen weiter destabilisieren.
- Das Erleiden bleibt weitgehend unsichtbar; strukturelle Probleme werden individualisiert.
- Um ein würdevolles Leben im Alter sicherzustellen, sind die beschriebenen Phänomene mehr in das Bewusstsein von Professionellen und Politik zu rücken sowie strukturelle Voraussetzungen zu deren Vermeidung bzw. Bewältigung zu schaffen.
